# Research on the Mechanism of Action of a Citrinin and Anti-Citrinin Antibody Based on Mimotope X27

**DOI:** 10.3390/toxins12100655

**Published:** 2020-10-13

**Authors:** Yanping Li, Yucheng Hu, Zhui Tu, Zhenqiang Ning, Qinghua He, Jinheng Fu

**Affiliations:** 1 State Key Laboratory of Food Science and Technology, Jiangxi-OAI Joint Research Institute, Jiangxi Province Key Laboratory of Modern Analytical Sciences, Nanchang University, Nanchang 330047, China; liyanping@ncu.edu.cn (Y.L.); tuzhui@ncu.edu.cn (Z.T.); heqinghua@ncu.edu.cn (Q.H.); 2 College of Food Science and Technology, Nanchang University, Nanchang 330047, China; Chengyh1173887145@163.com (Y.H.); zhenqiang-ning@seu.edu.cn (Z.N.)

**Keywords:** citrinin, mimotope, molecular docking, site-directed saturation, indirect competitive ELISA

## Abstract

Immunoassays are developed based on antigen–antibody interactions. A mimotope is an effective recognition receptor used to study the mechanism of action of antigens and antibodies, and is used for improving the sensitivity of the antibody. In this study, we built a 3D structure of the citrinin (CIT) mimotope X27 and anti-CIT single-chain antibody fragment (ScFv) through a “homologous modeling” strategy. Then, CIT and X27 were respectively docked to anti-CIT ScFv by using the “molecular docking” program. Finally, T28, F29, N30, R31, and Y32 were confirmed as the key binding sites in X27. Furthermore, the result of the phage-ELISA showed that the mutational phage lost the binding activity to the anti-CIT ScFv when the five amino acids were mutated to “alanine”, thereby proving the correctness of the molecular docking model. Lastly, a site-directed saturation strategy was adopted for the sites (T28, F29, N30, R31, and Y32). Eighteen different amino acids were introduced to each site on average. The activities of all mutants were identified by indirect competitive ELISA. The sensitivities of mutants T28F, T28I, F29I, F29V, N30T, and N30V were 1.83-, 1.37-, 1.70-, 2.96-, 1.31-, and 2.01-fold higher than that of the wild-type, respectively. In conclusion, the binding model between the CIT and antibody was elaborated for the first time based on the mimotope method, thereby presenting another strategy for improving the sensitivity of citrinin detection in immunoassays.

## 1. Introduction

Citrinin (CIT), a secondary fungal metabolite, is produced by several species of the genera *Penicillium*, *Aspergillus*, and *Monascus.* Citrinin can be detected from wheat, rye, corn, and rice [1,2,3]. Some animal experiments showed that citrinin has obvious toxic effects on the kidney, liver, gastrointestinal tract, and reproductive system; it can cause cell mutations, tumors, and carcinogenesis [4,5,6,7,8].

At present, citrinin detection methods include thin-layer chromatography [9], high-performance liquid chromatography [10], and liquid chromatography–tandem mass spectrometry (LC-MS) [11]. Thin-layer chromatography has the advantages of simple operation, low cost, and fast detection. However, this detection method has a low sensitivity and poor repeatability, and is not suitable for the accurate detection of samples in a batch. High-performance liquid chromatography has been widely used in the detection of citrinin and other mycotoxins because of its high sensitivity. However, it is time consuming, complicated, and expensive to operate. Moreover, it cannot adapt to the rapid detection of a large number of samples. The LC-MS method has the advantages of a high sensitivity and good repeatability, but it has the disadvantages of high cost and complex sample processing. Immunoassays have been widely performed for mycotoxin detection owing to its simplicity, specificity, and low cost [12,13,14].

Antigen–antibody interaction is the basis of immunodetection [15], and antibodies play a vital role. Such an interaction largely determines the sensitivity of the detection methods. Nanobodies are a kind of genetically engineered antibody that are modified through saturation, random mutagenesis, site-directed mutation, and rational design [16,17,18]. Nanobodies have the advantage of directed evolution. However, random mutagenesis is accompanied by randomness, heavy workload, and unsatisfied results [19]. Random mutations tend to destroy the stability of the protein [20]. The rational design of antibodies is a new strategy for improving the sensitivity of the antibodies proposed in recent years. The strategy has many advantages, such as saving time, low cost, and high success rate. Rational design is based on computer technology to simulate the 3D structure of the antibody molecules, and to predict the key binding site of the antigen–antibody. Then, the site is modified to enhance the affinity of the antibody [21,22].

Nanobodies are small in size, chemically stable, highly soluble, and easy to use. Furthermore, many researchers have proved that nanobodies are more suitable for the replacement of conventional antigens in immunoassays [23,24]. Qiu et al. developed an environmentally friendly immunoassay for the sensitive detection of the mycotoxin deoxynivalenol (DON) by using nanobodies as a DON mimotope [25]. Yang et al. explored the mechanism of interaction between the Ab42 antibody and its targeted antigen by molecular docking, molecular dynamics (MD) simulation, free-energy calculation, and computational alanine scanning (CAS) [26]. Luo et al. successfully obtained an anti-ricin chimeric monoclonal antibody with a sensitivity that is 13.5 times higher than that of the wild-type through homology modeling, molecular docking, and dynamics simulation methods [27]. Zhao et al. used aflatoxin B_1_ (AFB_1_) as a model system, and mimotopes of an aflatoxin nanobody Nb28 were screened by phage display. A rapid, magnetic, bead-based directed competitive ELISA (MB-dcELISA) was successfully developed by utilizing Nb28 and its mimotope ME17 [28].

In our previous work, we obtained a CIT mimotope from a naive alpaca, heavy-chain, single-domain antibody library [29]. Thirty random phage clones were selected after the fourth panning round and experimented with by phage ELISA. X27, the only clone, was considered as an anti-idiotype nanobody, which inhibited the binding of activity to the primary antibody by the free CIT standard [24]. We established a real-time immuno-PCR method for the quantitative determination of CIT based on mimotope X27. The IC_50_ value of the established method in the present study is 9.86 ± 2.52 ng/mL, which is nearly equivalent to that obtained when using the traditional phage-ELISA method [30]. The sensitivity of this method in detecting citrinin has not been significantly improved. However, the linearity range of the established method is 0.1–1000 ng/mL, which is 10-fold broader than that of the phage-ELISA method [30]. Here, we adopted another strategy to improve the sensitivity of the citrinin detection in immunoassays. The key binding sites of the citrinin mimotope X27 and anti-CIT single-chain antibody fragment (ScFv) were obtained by bioinformatics methods (molecular simulation and molecular docking). Then, site-directed mutations were performed on key sites to improve the sensitivity detection of CIT by indirect competitive ELISA. We preliminarily explained the binding mode of mimotope X27 and the anti-CIT antibody.

## 2. Results

### 2.1. Homologous Modeling and Molecular Docking

**Figure 1:**
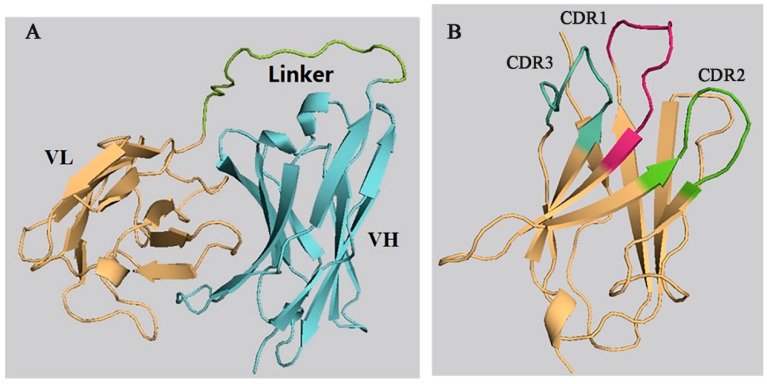
Prediction of the 3-D structure of the anti-CIT single-chain antibody fragment (ScFv) and X27. (**A**) anti-CIT ScFv; (**B**) X27.

**Figure 2:**
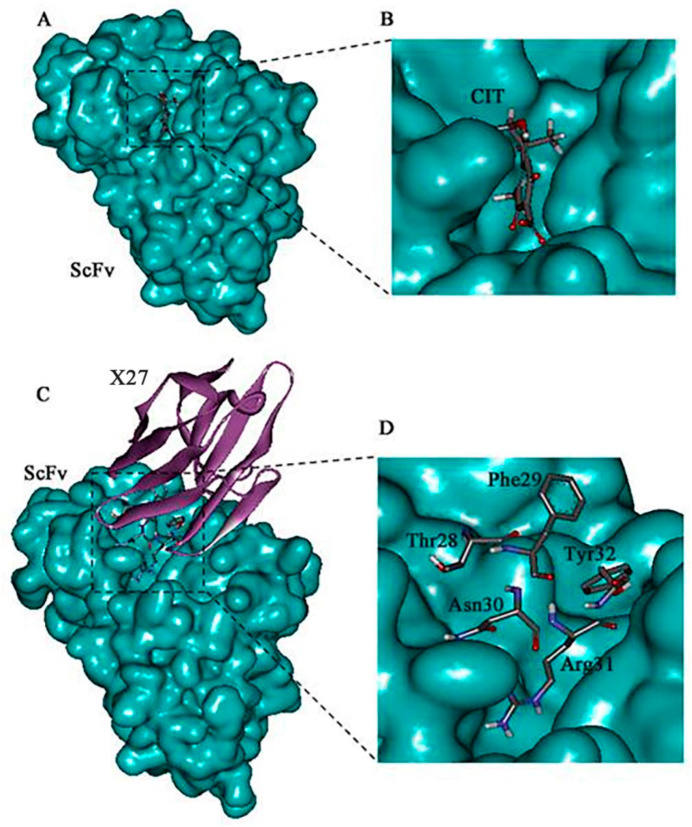
Molecular docking result of the CIT/ScFv and X27/ScFv. (**A**,**B**) Molecular modeling interaction between CIT and the anti-CIT ScFv. (**C**,**D**) Molecular modeling interaction between X27 and the anti-CIT ScFv. The structure of the anti-CIT ScFv is shown in blue color, the structure of X27 in eggplant color, and the binding site domain (amino acids 28–31) in gray.

The 3-D models of the anti-CIT ScFv and mimotope X27 were generated by using SWISS-MODEL. The anti-CIT ScFv is composed of linker (the green), heavy-chain variable regions (the cyan), and light-chain variable regions (the yellow). Its framework region is composed of antiparallel β-sheets. The mimotope X27 framework region, composed of the anti-parallel β-sheet structure, is an irregular coil and an α-helix structure. The CDR regions are distributed at the N-terminus of mimotope X27, and the CDR1, CDR2, and CDR3 regions were marked in red, green, and cyan, respectively (Figure 1). Then, the quality of the 3-D models was assessed by a Ramachandran plot, and the above 95% residues of the anti-CIT ScFv and mimotope X27 model were in a favorable region (Figure S1). Molecular docking of CIT and mimotope X27 with the anti-CIT ScFv indicated that both X27 and CIT can interact with the Tyr33, Leu100, and Ser183 of the anti-CIT ScFv. There exist hydrogen bond interactions between the hydrogen atom on the carboxyl group of CIT and Leu100 of the anti-CIT ScFv (data not shown). At least four hydrogen bonds also were found between X27 and the anti-CIT ScFv (X27: Thr28-ScFv: Tyr33; X27: Thr28-ScFv: Asp52; X27: Arg31-ScFv: Leu100; X27: Tyr32-ScFv: Glu59) (Figure 2).

The mimotope X27 (Arg31) and CIT recognize the same region and hotspot residue Leu100 of the anti-CIT ScFv, explaining the rational of using mimotope X27 for immunological analysis. Residue F29 and N30 in X27 are located in the center of the active binding site of the anti-CIT ScFv, and the two residues may participate in the antigen–antibody interaction. Therefore, the five above mentioned amino acids (T28, F29, N30, R31, and Y32) were identified as the key sites for the interaction of X27 and the anti-CIT ScFv.

### 2.2. Docking Model Verification

**Figure 3:**
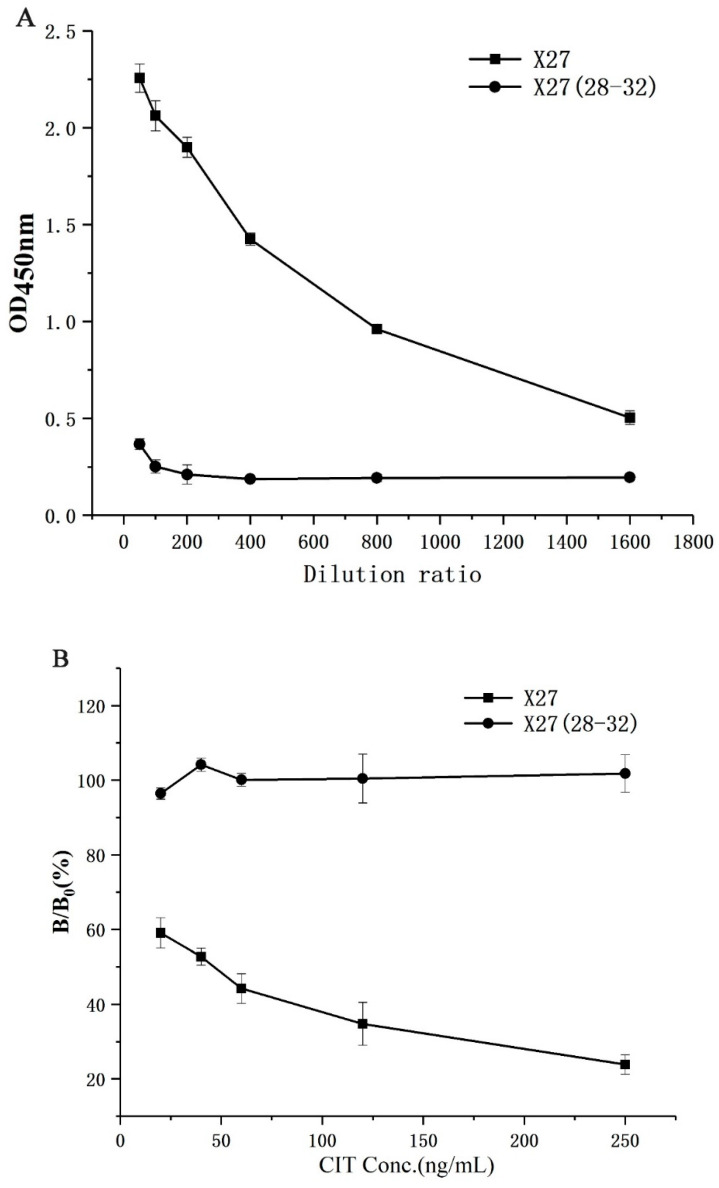
Identification of activity of X27 (28–32): (**A**) binding activity; (**B**) competitive activity. Each value is the average of three independent experiments.

In order to verify the correctness of the docking model, X27 (28–32) was constructed through the five amino acids 28–32 in the WT, mutated to T28AF29AN30AR31AY32A, and the binding activity of the mutant was identified by indirect competitive ELISA. As a result, the wild-type phage had a greater binding ability of the anti-CIT monoclonal antibody than X27 (28–32) at the same titer. Moreover, the X27 (28–32) phages basically lost the binding ability to CIT MAb (monoclonal antibody) when both the X27 and X27 (28–32) phages were diluted by more than 400 times (Figure 3A). The above mentioned concentrations of the X27 and X27 (28–32) phages were diluted 400 and 2 times, respectively, and indirect competitive ELISA was performed with different concentrations of CIT standards. The binding CIT MAb rate of the wild-type X27 gradually decreased linearly in the presence of CIT, but X27 (28–32) showed no obvious change (Figure 3B). Based on the above results, the wild-type X27 would gradually lose its binding ability to anti-CIT MAb and the competition ability of CIT when the amino acids 28–32 of the wild-type X27 were mutated to alanine at the same time. Therefore, the 28–32 amino acids of wild-type X27 can successfully be used to prove the key sites for interaction with anti-CIT MAb, and accurately reflected the binding patterns of mimotope X27 and anti-CIT MAb.

### 2.3. Alanine Scan

Alanine scanning was performed to confirm the role of each amino acid (28–32- and 74-site amino acids) after molecular docking and ELISA experiments to determine the key action sites in mimotope X27.

Primer design (Table 1) and cloning vector construction were performed for each site. Colony PCR verification was adopted on the clones obtained after transformation. The PCR bands of all the clones indicated that the target fragment was successfully inserted into the pHEN-1 vector. Furthermore, all clones successfully introduced the alanine mutations at the target site after sequencing by Genscript (Nanjing, China).

**Figure 4:**
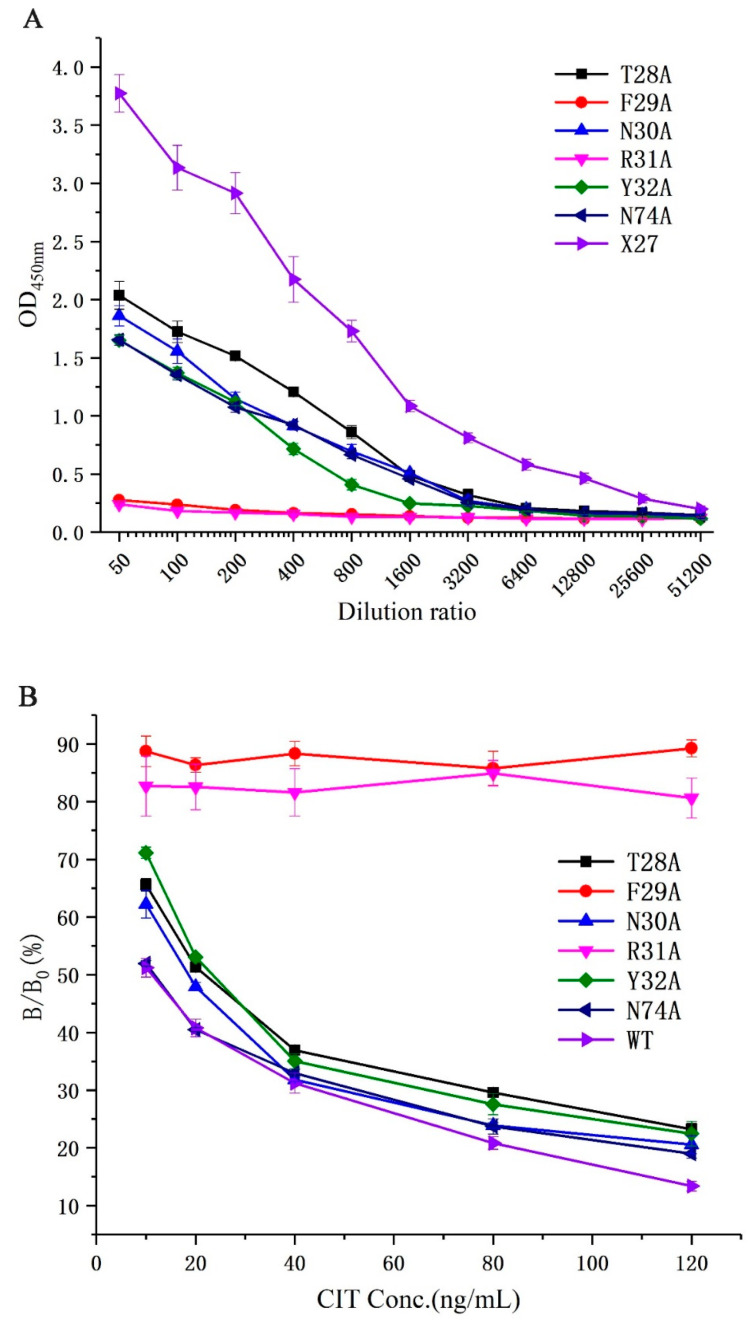
(**A**) Analysis of the binding activity of the X27 mutants. (**B**) Analysis of the competitive activity of the X27 mutants to CIT. Each value is the average of three independent experiments.

All phages (T28A, F29A, N30A, R31A, Y32A, N74A, and X27) were diluted to 1.2 × 10^13^ pfu/mL as the initial concentration to determine their binding activities to monoclonal antibodies and the competitive activity of the CIT standard through phage-ELISA. The binding activity of all mutants decreased compared with that of the wild-type X27 when the amino acids (28–32, and 74) were mutated to alanine. Furthermore, the mutants F29A and R31A decreased significantly and lost binding activity. The mutants T28A, N30A, Y32A, and N74A had about a 10-fold lower binding activity than the wild-type X27. (Figure 4A). This finding suggests that the amino acids at positions 28–31 and 74 in mimotope X27 are involved in the binding activity, among which the amino acids 29 and 31 play a vital role. In addition, we performed indirect competitive ELISA experiments on X27 and its mutants with different concentrations of CIT standards. The competitive activity of the X27 mutants (T28A, N30A, Y32A, and N74A) to CIT decreased with increasing CIT standard concentrations, but the mutants F29A and R31A showed no obvious change (Figure 4B).

We can infer that the amino acids (29- and 31-site amino acid) of X27 play an indispensable role in the binding of anti-CIT MAb. The mutants (T28A, N30A, and Y32A) indicated that the 28-, 30-, and 32-site amino acids of X27 play a minor auxiliary role in the binding of anti-CIT MAb compared with wild-type X27. The activity of mutant N74A and wild-type X27 seemed to be the same. Thus, we can conclude that the 74-site amino acid had no effect on the binding of anti-CIT MAb. This result lays the experimental foundation for further investigation on the binding mechanism of mimotope X27 and anti-CIT MAb.

### 2.4. Generation and Screen of Site-Directed Saturation Mutants

**Figure 5:**
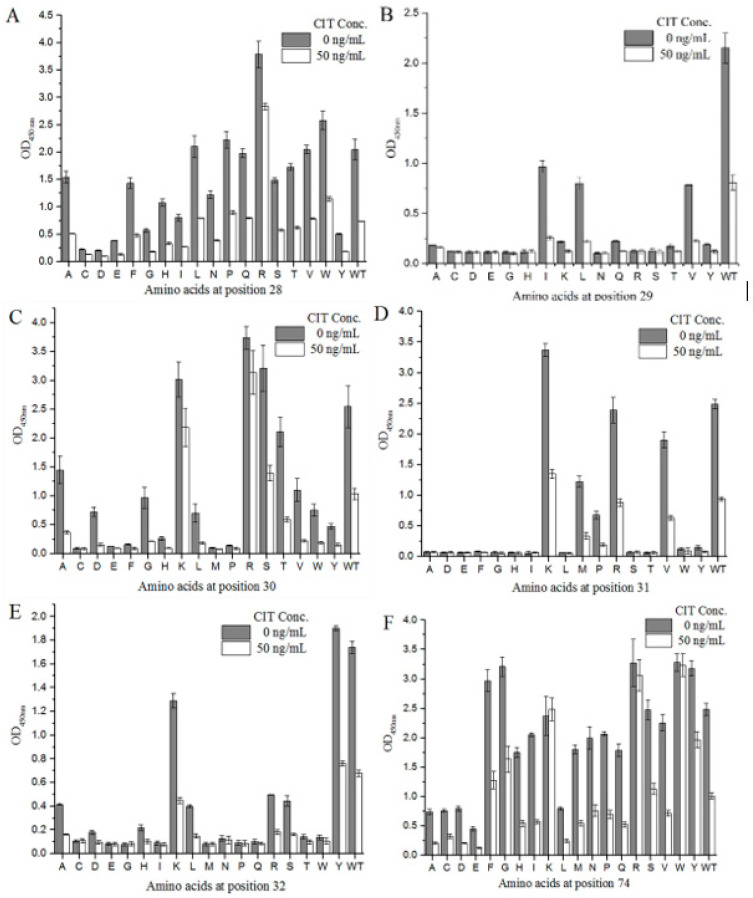
Identification of the activity of the X27-based, site-directed saturation transformants: (**A**–**E**) position 28–32; (**F**) position 74. Each value is the average of three independent experiments.

We designed degenerate primers to conduct site-directed saturation mutation experiments on mimotope X27 (28–32 and 74). Using M13R and pHEN-R as the specific primers, a single target fragment was successfully amplified with the correct band size (data not shown). All clones inserted the target fragment. Sixty positive clones were selected from each group (T28, F29, N30, R31, Y32, and N74) and sent for sequencing. The sequencing results were grouped at different positions (28–32- and 74-site amino acids), and the sequences of each group were compared using DNASTAR software. As a result, 18 different amino acids were introduced at each position on average. The X27 phage was appropriately diluted, so that the OD_450 nm_ of the indirect competitive ELISA was around 2.0, and the X27 phage titer was 2.4 × 10^11^ pfu/mL. All the cloned phages to be identified were diluted with the above X27 phage titer of 2.4 × 10^11^ pfu/mL to perform the indirect competitive ELISA. When the six sites (28–32- and 74-site amino acids) were mutated to other amino acids, the affinity and sensitivity of the phage changed differently (Figure 5). When the amino acids at positions 29 and 32 were mutated, the mutants mostly showed a weakened affinity and sensitivity, indicating that the two sites played a key role in the specific binding of mimotope X27 and anti-CIT MAb. At position 31, the K mutation shows a better affinity compared to the WT, but R31K showed lower inhibition rates than the WT(R). Therefore, the amino acid at position 31 is also considered to be the key binding site.

### 2.5. Establishment of the Standard Curve

**Figure 6:**
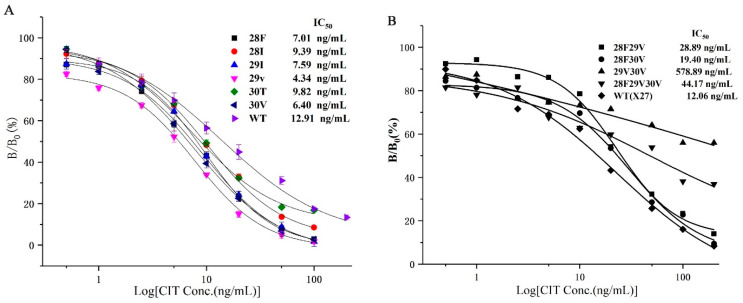
(**A**) The standard curve of the seven clones for CIT analysis by phage-ELISA. (**B**) The standard curve of the random assortment mutants. Each value is the average of three independent experiments.

Combining the affinity and sensitivity of the antibody to determine the optimal clones (Figure 5), we selected the six optimal clones (T28F, T28I, F29I, F29V, N30T, and N30V) based on the site-saturated mutation experiment, and prepared the CIT indirect competitive ELISA standard curve. The IC_50_ values of the six mutations and wild-type X27 were compared. The IC_50_ values of the six mutants were lower than that of wild-type X27. The IC_50_ values of the T28F, T28I, F29I, F29V, N30T, and N30V mutants reached 7.01, 9.39, 7.59, 4.34, 9.82, and 6.40 ng/mL, respectively. The sensitivity of the F29V mutant was 2.96 times higher than that of the wild-type X27 (12.92 ng/mL) (Figure 6A).

## 3. Discussion

### 3.1. Constructing a Site-Saturated Mutation Library to Screen Mutants

We constructed a site-saturated mutation library to screen mutants with increased sensitivity (date not shown) after identifying five key sites through alanine scanning. First, the designed degenerate primers were used to introduce random mutations, and the site-specific saturated mutation library was acquired according to the mature method of constructing phage display libraries in our laboratory. We randomly selected 24 clones from the transformed 3.6 × 10^7^ positive clones and performed colony PCR to verify the insertion rate of the mutant fragments. All 24 clones correctly inserted the mutant fragments, and the positive rate was 100%. Moreover, the sequences of No. 4 and No. 9 were the same after sequencing the abovementioned 24 clones, and the diversity of the sequences reached 96% (Figure S2). Therefore, the library capacity of the constructed library was 3.45 × 10^7^ (initial library capacity × positive rate × diversity) (theoretical library capacity, nucleic acid level: 4^10^ × 2^5^ = 3.36 × 10^7^; amino acid level: 20^5^ = 3.2 × 10^6^). The constructed site-saturated mutation library theoretically covered the possibility of all mutations in terms of diversity. Thus, it can be used as a mutation library to screen the improved sensitivity of mutants. Ninety-six clones were randomly selected from the eluted phages of 2, 3, and 4 rounds for indirect competitive ELISA identification after 4 rounds of screening. However, the results showed that the clones were not screened for improved sensitivity compared with the wild-type X27. This result may be due to the large base of mutant diversity in the site-saturated mutation library, and termination codons were introduced into the mutational sites. Thus, few clones with improved sensitivity from such a rich library are difficult to select. Therefore, we finally adopted the method of single-point saturation mutation to screen for mutants with improved sensitivity.

### 3.2. Mutant F29V Sensitivity Improvement Mechanism

In this study, the mutant F29V was obtained based on the mimotope X27, combined with rational design strategies and molecular biological methods, and the IC_50_ value of the indirect competitive ELISA method based on X27 was reduced 2.96 times. The decrease in IC_50_ means that the affinity of the mock antigen to the antibody is reduced [31], which is the reason why the mutant F29V had a lower affinity to the anti-CIT ScFv than the wild-type X27. Comparing the amino acids between the mutants and wild-type X27, no significant difference was found in amino acid properties, because phenylalanine and valine are non-polar amino acids. However, phenylalanine has one more benzene ring compared with valine in terms of molecular structure. The π-π stacking interaction may be formed by the benzene ring of phenylalanine and the aromatic ring of tyrosine. The tyrosine is located at position 33 of the single-chain antibody (Figure S3C). Thus, the decrease in affinity is due to the disappearance of the π-π interaction in F29V. In addition, we docked the 3D structure of F29V with the anti-CIT ScFv. The data showed that the overall structure of the mimotope and the local CDR regions did not have an obvious impact when the amino acid was mutated from phenylalanine to valine at position 29 (Figure S3). However, the analysis of hydrogen-bonding on the antigen–antibody binding surface revealed that X27 has eight hydrogen-bonding interactions with single-chain antibodies, while F29V has only seven (Table 2). Therefore, it can be presumed that the reduction in hydrogen bonding is also one of the main reasons for the lower affinity between F29V and the anti-CIT antibody [32].

The lower the affinity of the mimotope to the antibody, the higher the sensitivity of the immunological analysis method will be. In this study, RNA was extracted from anti-CIT MAb hybridoma cells to obtain the sequence of single-chain antibodies that were used for homology modeling and to obtain the 3D structure. Then, we separately docked CIT and mimotope X27 with the anti-CIT ScFv by using molecular docking software. CIT and mimotope X27 were successfully docked in the same active pocket located between the heavy-chain variable region and the light-chain variable region of the single-chain antibody. This finding explains the mechanism of mimotope X27 replacing artificial antigens in immunoassay methods from a molecular mechanism perspective.

### 3.3. Random Combination of Optimal Mutation Sites for T28F, F29V, and N30V

Six clones, namely, T28F, T28I, F29I, F29V, N30T, and N30V, were selected from the site-saturated mutant clones, and sensitivities were increased by 1.83, 1.37, 1.70, 2.96, 1.31, and 2.01 times compared with that of the wild-type, respectively. Subsequently, the optimal random combination of amino acids was used to study whether the sensitivity of the immunological analysis method could be improved again, so we constructed four plasmids: pHEN-X27-28F29V, pHEN-X27-28F30V, pHEN-X27-29V30V, and pHEN-X27-28F29V30V. However, the sensitivities of the mutants 28F29V, 28F30V, and 28F29V30V were all reduced compared with that of the wild-type X27 through an indirect competitive ELISA (Figure 6B). The sensitivity of the immunological analysis methods has decreased due to the improved binding ability of the mimotope to the monoclonal antibody after the optimal amino acid combination.

## 4. Conclusions

This study was based on the CIT mimotope X27, which was firstly modified by genetic engineering to investigate the mechanism of recognition of CIT and anti-CIT antibody. It improved the sensitivity of the immunological analysis method to detect CIT. The 3D structures of the anti-CIT ScFv and mimotope X27 were constructed by homology modeling strategies, respectively. We successfully obtained key sites, T28, F29, N30, R31, and Y32, which are involved in the antigen–antibody reaction, after using CIT and mimotope X27 to dock with CIT single-chain antibodies. Furthermore, the five key sites, T28, F29, N30, R31, and Y32, were the five active sites of CIT and the anti-CIT ScFv, as proved by alanine mutation and indirect competitive ELISA. Then, site-directed saturation was performed on the six key sites, namely, T28, F29, N30, R31, Y32, and N74. We finally screened the mutants (T28F, T28I, F29I, F29V, N30T, and N30V) with a higher sensitivity than the wild type. Moreover, the IC_50_ value of F29V reached 4.34 ng/mL, which was 2.96 times higher than that of the wild-type X27.

## 5. Materials and Methods

### 5.1. Bacterial Strains, Plasmids, Libraries, and Helper Phage and Reagents

Citrinin standard was purchased from Sigma (St. Louis, MO, USA). Restriction enzymes *Nco* I and *Not* I and T4 DNA ligase were purchased from TaKaRa (Dalian, China). Helper phage M13KO7 was a generous gift from Dr. Wei-Jun Ma (Shanghai Institutes for Biological Sciences, Chinese Academy of Sciences). Mimotope X27 and the citrinin antibody were previously prepared in our laboratory [24].

### 5.2. Homologous Modeling and Molecular Docking

The amino acid sequences of the anti-CIT ScFv and mimotope X27 were uploaded to the official website of SWISS-MODEL (https://www.swissmodel.expasy.org/) to predict the 3D structure [33]. The 3D structures of the single-chain antibody and mimotope X27 were obtained using three crystal structures with high homology as templates, respectively. We evaluated the highest score 3D structure by a Ramachandran plot. We built the 3D structure of CIT through the website (https://pubchem.ncbi.nlm.nih.gov/, PubChem CID: 54680783). All templates were done energy minimized and equilibrated before molecular docking. In the molecular docking, the Euler angle was 6° and the other parameters were set as the default. When evaluating the poses that were obtained by molecular docking, the pose with the highest score was the optimal docking model. Finally, CIT and X27 were docked to the anti-CIT ScFv using ZDOCK molecular docking software [34].

### 5.3. Validation of Molecular Docking

Plasmid pHEN-X27 was used as a template. Two pairs of primers (223 and 226, and 224 and 255) (Table 1) were used to amplify the fragments of A and B. The fragments of A and B, which contain the full length of the mutant gene, were used as templates (primer pairs 223 and 224, respectively). Each PCR (50 μL) involved 100 ng of plasmid, 2.5 mM dNTP, 10 μM primers, 10 μL of 5× reaction buffer, and 0.5 μL of PrimerSTAR enzyme. The reaction was performed at 95 °C for 3 min, followed by 30 cycles of 95 °C for 30 s, 55 °C for 30 s, 72 °C for 30 s, and then 72 °C for 10 min. The purified mutant X27 (28-32) and phagemid pHEN-1 were digested with restriction enzymes *Nco* I and *Not* I, respectively. Afterward, the validated X27 (28-32) and phagemid pHEN-1 were ligated and transformed into TG1 competent cells by heat shock at 42 °C for 90 s [35], and cultured in LB medium (containing 100 mg/mL ampicillin) at 37 °C. Next, the positive clones were selected and sequenced. Finally, phage ELISA was used to identify phage activity, and wild-type X27 was used as the positive control. The X27 and X27 (28–32) phages were diluted to 1.2 × 10^13^ pfu/mL with PBS to determine the binding activity. Anti-CIT monoclonal antibody was coated into the wells of a 96-well microplate and two phages with different dilutions for ELISA were added.

### 5.4. Alanine Scan

The wild-type amino acids 28–32 and 74 (the changes of the 74-site amino acid will affect the sensitivity of X27, data not shown) were individually alanine mutated, and the binding activity of the mutant was identified using phage-ELISA (its details were available in a previous step, and for the primers see Table 1). The CIT standard concentrations were 10, 20, 40, 80, and 120 ng/mL.

### 5.5. Generation and Screen of Site-Directed Saturation Mutants

For construction of the 28–32- and 74-site amino acids, site-saturated mutant clones, using an overlapping extension PCR and degenerate primers (see Table 1), were used to randomly introduce different bases. After the colony PCR verified that the insertion rate of the target fragment was greater than 90%, 60 randomly selected positive clones from each group were sequenced for identification, and clones with different amino acid sequences were selected for identification. Experimental step: anti-CIT MAb ascites were diluted 1000-fold with 1 × PBS, 100 μL was added to the microtiter plate, and coated overnight at 4 °C. Following washing 3 times with PBST, the wells were blocked with 4% skim milk in PBS at 37 °C for 1 h. After the wells were washed 3 times with PBST, 50 μL of appropriate phage solution and 50 μL of 50 ng/mL the CIT standard were added and incubated for 1 h. After the wells had been washed 4 times with PBST, 100 μL of anti-M13 phage antibody (1:5000 diluted in PBS) was added and incubated for 30 min. Then, 100 μL of 3,3′,5,5′-tetramethylbenzidine (TMB) substrate was added to the washed wells and incubated for 6 min. Finally, the reaction was stopped by adding 50 μL of 2 M H_2_SO_4_ and the absorbance of 450 nm was detected by microplate reader (Thermo Scientific, Waltham, MA, USA).

### 5.6. Establishment of Standard Curve

The standard curve was established for the improved sensitivity mutant clones. We used the checkerboard titration method to optimize the monoclonal antibody coating amount and phage input amount for establishing the standard curve of each mutant indirect competitive ELISA. The wells of a 96-well microplate were coated with 100 μL of 1.5 μg/mL anti-CIT MAb overnight at 4 °C and blocked with 4% skim milk in PBS for 1 h at 37 °C. Following washing 3 times with PBST, 50 μL of appropriate phage solution and 50 μL of 50 ng/mL the CIT standard at various concentrations were added and incubated for 1 h. After the wells had been washed 4 times with PBST, 100 μL of anti-M13 phage antibody (1:5000 diluted in PBS) was added and incubated for 30 min. Then, 100 μL TMB substrate was added to the washed wells and incubated for 30 min. Finally, the reaction was stopped by adding 50 μL of 2 M H_2_SO_4_ and the absorbance of 450 nm was detected by microplate reader.

## Figures and Tables

**Table 1:** The primers used in this study.

Numbers	Primers	Sequences (5′→3′)
223	M13-r	AGCGGATAACAATTTCACACAGGA
224	pHEN-r	GCCCCATTCAGATCCTCTTC
225	(28-32)A-f	TCTGGACGCGCTgctgctgctgctCCCATGAGCTGGTTCCGCC
226	(28-32)A-r	CTCATGGGagcagcagcagcagcGCGTCCAGAGCCTGCACAGG
230	T28A-f	TCTGGACGCgctTTCAATAGGTATCCCATG
231	T28A-r	CCTATTGAAagcGCGTCCAGAGCCTGCACA
232	F29A-f	GGACGCACCgctAATAGGTATCCCATGAGC
233	F29A-r	ATACCTATTagcGGTGCGTCCAGAGCCTGC
234	N30A-f	CGCACCTTCgctAGGTATCCCATGAGCTGG
235	N30A-r	GGGATACCTagcGAAGGTGCGTCCAGAGCC
236	R31A-f	ACCTTCAATgctTATCCCATGAGCTGGTTC
237	R31A-r	CATGGGATAagcATTGAAGGTGCGTCCAGA
238	Y32A-f	TTCAATAGGgctCCCATGAGCTGGTTCCGC
239	Y32A-r	GCTCATGGGagcCCTATTGAAGGTGCGTCC
240	N74A-f	TCCAGAGACgctGCCAAGAACACGGTGTTT
241	N74A-r	GTTCTTGGCagcGTCTCTGGAGATGGTGAA
245	T28ss-f	TCTGGACGCnnkTTCAATAGGTATCCCATG
246	T28ss-r	CCTATTGAAmnnGCGTCCAGAGCCTGCACA
247	F29ss-f	GGACGCACCnnkAATAGGTATCCCATGAGC
248	F29ss-r	ATACCTATTmnnGGTGCGTCCAGAGCCTGC
249	N30ss-f	CGCACCTTCnnkAGGTATCCCATGAGCTGG
250	N30ss-r	GGGATACCTmnnGAAGGTGCGTCCAGAGCC
251	R31ss-f	ACCTTCAATnnkTATCCCATGAGCTGGTTC
252	R31ss-r	CATGGGATAmnnATTGAAGGTGCGTCCAGA
253	Y32ss-f	TTCAATAGGnnkCCCATGAGCTGGTTCCGC
254	Y32ss-r	GCTCATGGGmnnCCTATTGAAGGTGCGTCC
255	N74ss-f	TCCAGAGACnnkGCCAAGAACACGGTGTTT
256	N74ss-r	GTTCTTGGCmnnGTCTCTGGAGATGGTGAA

m = A/C; n = A/T/G/C; k = G/T; lowercase letters represent pseudo-mutation sites.

**Table 2:** Hydrogen bonds observed between the anti-CIT ScFv and X27 or F29V.

Numbers	X27: Anti-CIT ScFv	F29V: Anti-CIT ScFv
1	Thr28:Tyr33	Thr28:Tyr33
2	Ala75:Arg102	Ala75:Arg102
3	Gln1:Asn55	Gln1:Asn55
4	Thr28:Asp52	Thr28:Asp52
5	Arg31:Leu100	Arg31:Leu100
6	Tyr32:Glu59	Tyr32:Glu59
7	Trp53:Ser183	Trp53:Ser183
8	Try106:Asp57	

## Supplementary Materials

The following are available online at https://www.mdpi.com/2072-6651/12/10/655/s1, Figure S1: The Ramachandran Plot of the models of anti-CIT ScFv (A) and X27 (B). Cyan region: Hardsphere, Magenta region: Overlap. Figure S2: Sequence analysis of 24 clones from Site-directed saturation library. Figure S3: The Docking results of anti-CIT ScFv (A) and F29V-ScFv (B). (C) An enlarged view of the dotted box part in panel A.
