# Supplementary material for: Research on the Mechanism of Action of a Citrinin and Anti-Citrinin Antibody Based on Mimotope X27
Source: Toxins (Basel). 2020 Oct 13;12(10):655. doi: 10.3390/toxins12100655 (PMC7602013; doi:10.3390/toxins12100655)
Supplement: Supplementary file 1 [file toxins-12-00655-s001.pdf]

Supplementary Materials: Research on the Mechanism of Action of Citrinin and Anti- Citrinin Antibody Based on Mimotope X27

Yanping Li, Yucheng Hu, Zhui Tu, Zhenqiang Ning, Qinghua He and Jinheng Fu

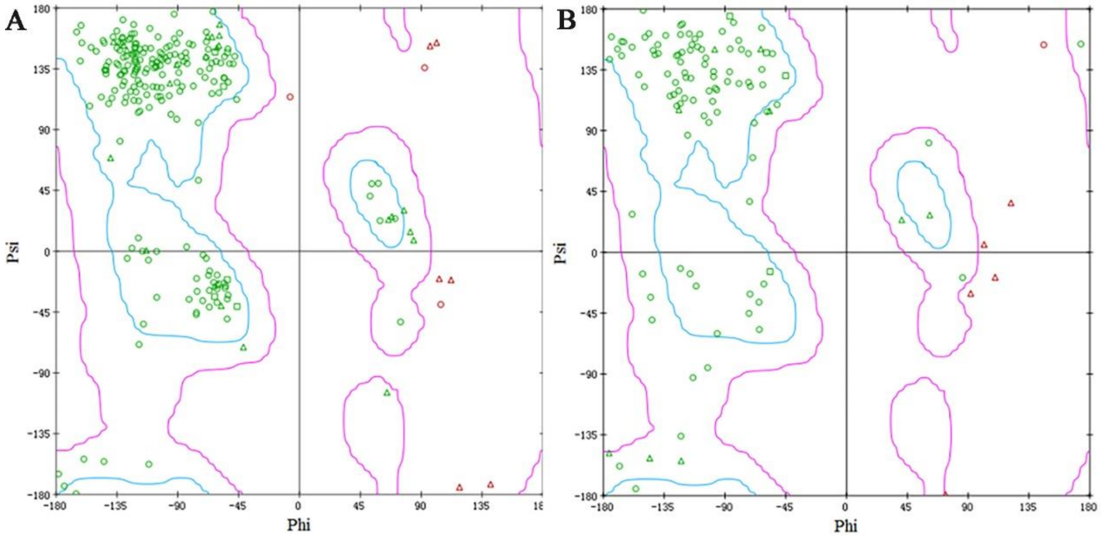

Figure S1. The Ramachandran Plot of the models of anti-CIT ScFv(**A**) and X27(**B**). Cyan region: Hardsphere, Magenta region: Overlap.

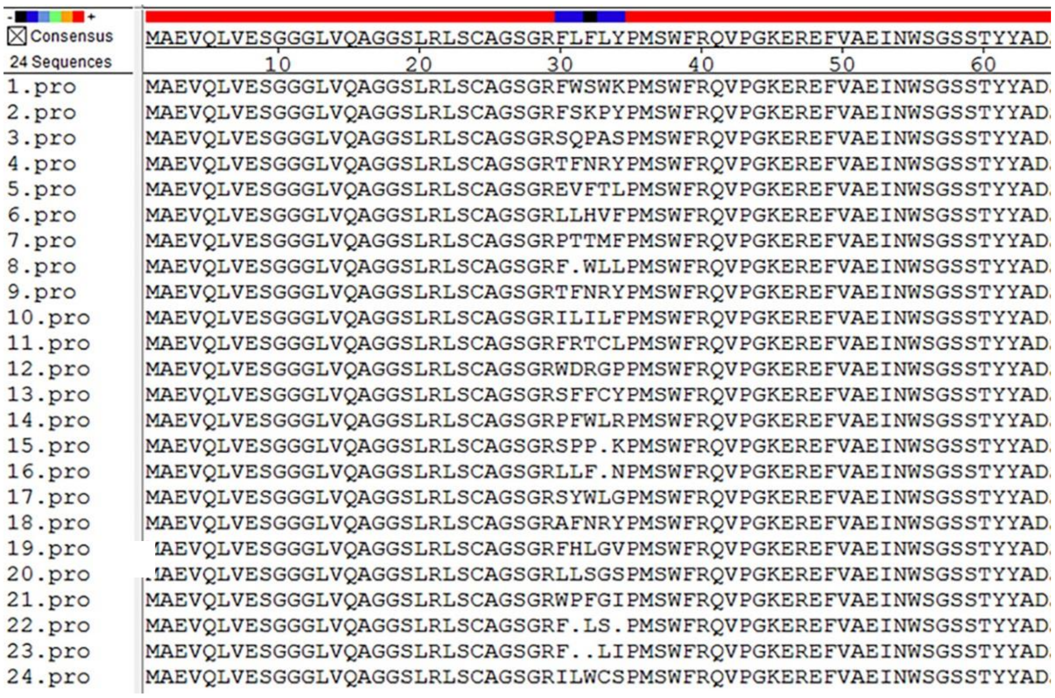

Figure S2. Sequence analysis of 24 clones from Site-directed saturation library.

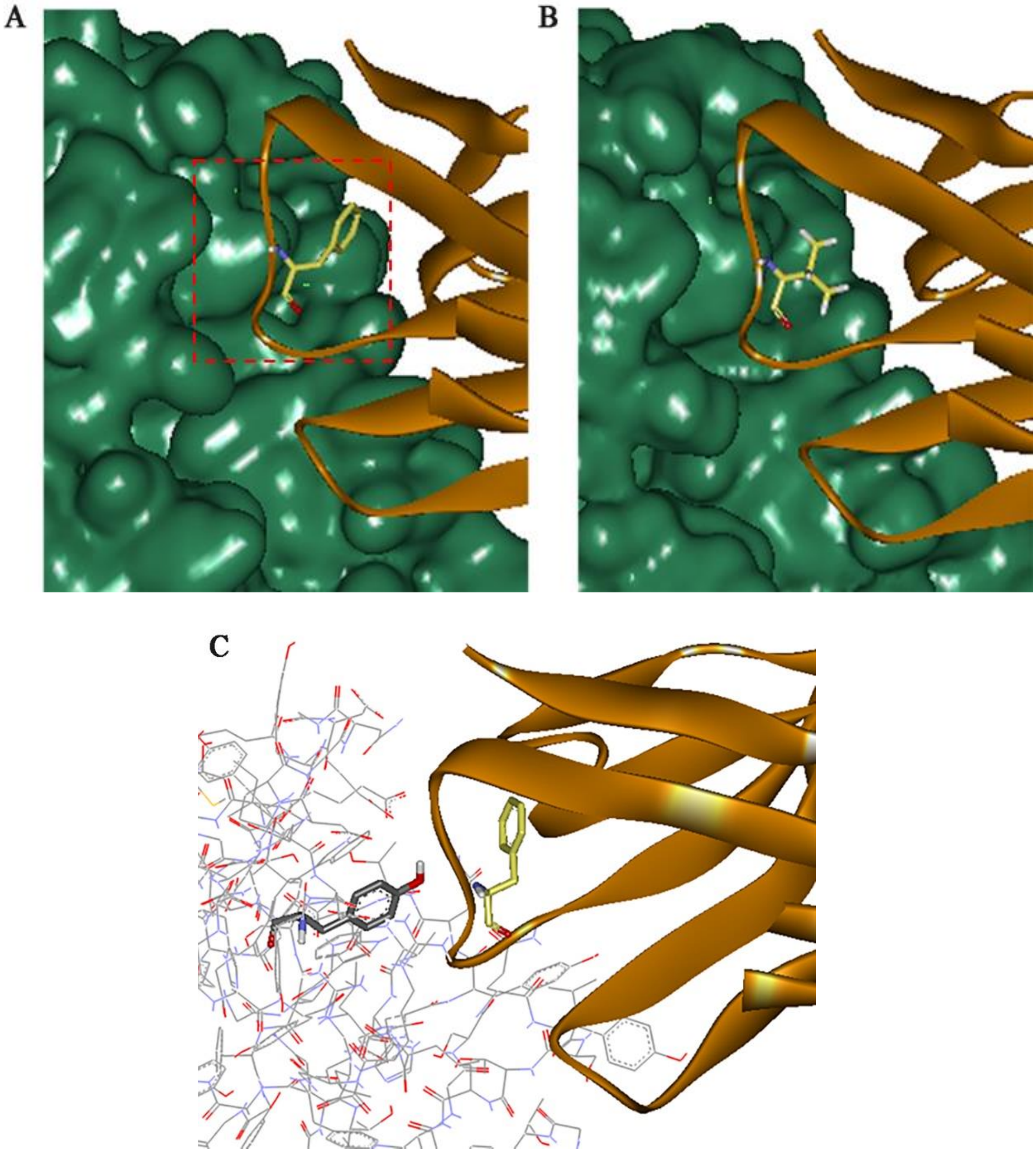

Figure S3. The Docking results of anti-CIT ScFv(**A**) and F29V-ScFv(**B**). (**C**) An enlarged view of the dotted box part in panel A.
